# Supplementary material for: Meaning in Life and the Acceptance of Cancer: A Systematic Review
Source: Int J Environ Res Public Health. 2022 May 3;19(9):5547. doi: 10.3390/ijerph19095547 (PMC9104184; doi:10.3390/ijerph19095547)
Supplement: Supplementary file 1 [file ijerph-19-05547-s001.zip › ijerph-1661239-supplementary.pdf]

International Journal of
Environmental Research and Public Health

MDPI

Table S1. Syntax for search strategy.

Database	Search Strategy
**Medline**	((MH "Neoplasms") OR (TI cancer* OR AB cancer*) OR (TI tumo#r OR AB tumo#r) OR (TI oncolog* OR AB oncolog*) OR (TI sarcoma* OR AB sarcoma*) OR (TI carcinoma* OR AB carcinoma*) OR (TI neoplas* OR AB neoplas*)) AND ((MH "Spirituality") OR (MH "Resilience, psychological") OR (MH "Self Concept") OR (TI "Purpose in life" OR AB "Purpose in life") OR (TI "Life purpose" OR AB "Life purpose") OR (TI "Sense of purpose" OR AB "Sense of purpose") OR (TI "Sense of coherence" OR AB "Sense of coherence") OR (TI "Meaning-making" OR AB "Meaning-making") OR (TI "Meaning making" OR AB "Meaning making") OR (TI "Meaning in life" OR AB "Meaning in life") OR (TI "Finding meaning" OR AB "finding meaning") OR (TI "Meaning of life" OR AB "Meaning of life") OR (TI "Sense of meaning" OR AB "Sense of meaning") OR (TI "Life meaning" OR AB "Life meaning") OR (TI "Search for meaning" OR AB "Search for meaning") OR (TI "Personal meaning" OR AB "Personal meaning") OR (TI "MEANINGFULNESS" OR AB "MEANINGFULNESS") OR (TI "Self-trascendence" OR AB "Self-trascendence") OR (TI "Global belief*" OR AB "Global belief*") OR (TI "Existential" OR AB "Existential") OR (TI "Spiritual well-being" OR AB "Spiritual well-being") OR (TI "Life engagement" OR AB "Life engagement") OR (TI "Evaluation of life" OR AB "Evaluation of life") OR (TI "Value of life" OR AB "Value of life")) AND ((MH "Adaptation, psychological") OR ("Accept* of illness" OR "Accept* of diagnosis" OR "Accept* of cancer" OR "Accept* of disease" OR "Accept* illness" OR "Accept* diagnosis" OR "Accept* cancer" OR "Accept* disease" OR "Illness accept*" OR "Diagnosis accept*" OR "Cancer accept*" OR "Disease accept*" OR "Adjust* to illness" OR "Adjust* to diagnosis" OR "Adjust* to cancer" OR "**Adjust** to disease" OR "Illness adjust*" OR "Diagnosis adjust*" OR "Cancer adjust*" OR "Disease adjust*" OR "Adapt* to illness" OR "Adapt* to diagnosis" OR "Adapt* to cancer" OR "Adapt* to disease" OR "Illness adapt*" OR "Diagnosis adapt*" OR "Cancer adapt*" OR "Disease adapt*" OR "Mak* peace" OR "Peace with illness" OR "Peace with diagnosis" OR "Peace with cancer" OR "Peace with disease" OR "Peace* accept*"))
**PsychInfo**	((DE "Neoplasms") OR (DE "Benign Neoplasms") OR (DE "Breast Neoplasms") OR (DE "Endocrine Neoplasms") OR (DE "Melanoma") OR (DE "Metastasis") OR (DE "Leukemias") OR (DE "Nervous System Neoplasms") OR (DE "Terminal Cancer") OR (TI cancer* OR AB cancer*) OR (TI tumo#r OR AB tumo#r) OR (TI oncolog* OR AB oncolog*) OR (TI sarcoma* OR AB sarcoma*) OR (TI carcinoma* OR AB carcinoma*) OR (TI neoplas* OR AB neoplas*)) AND ((DE "Meaning") OR (DE "Spirituality") OR (DE "Resilience Psychological) OR (DE "Self Concept") OR (TI Purpose in life OR AB Purpose in life) OR (TI Life purpose OR AB Life purpose) OR (TI Sense of purpose OR AB Sense of purpose) OR (TI Sense of coherence OR AB Sense of coherence) OR (TI "Meaning-making" OR AB "Meaning-making") OR (TI "Meaning making" OR AB "Meaning making") OR (TI "Meaning in life" OR AB "Meaning in life") OR (TI "Finding meaning" OR AB "finding meaning") OR (TI "Meaning of life" OR AB "Meaning of life") OR (TI "Sense of meaning" OR AB "Sense of meaning") OR (TI "Life meaning" OR AB "Life meaning") OR (TI "Search for meaning" OR AB "Search for meaning") OR (TI "Personal meaning" OR AB "Personal meaning") OR (TI "MEANINGFULNESS" OR AB "MEANINGFULNESS") OR (TI "Self-trascendence" OR AB "Self-trascendence") OR (TI Global belief* OR AB Global belief*) OR (TI Existential OR AB Existential) OR (TI "Spiritual well-being" OR AB "Spiritual well-being") OR (TI Life engagement OR AB Life engagement) OR (TI Evaluation of life OR AB Evaluation of life) OR (TI Value of life OR AB Value of life)) AND ((DE "Emotional Adjustment") OR ("Accept* of illness" OR "Accept* of diagnosis" OR "Accept* of cancer" OR "Accept* of disease" OR "Accept* illness" OR "Accept* diagnosis" OR "Accept* cancer" OR "Accept* disease" OR "Illness accept*" OR "Diagnosis accept*" OR "Cancer accept*" OR "Disease accept*" OR "Adjust* to illness" OR "Adjust* to diagnosis" OR "Adjust* to cancer" OR "Adjust to disease" OR "Illness adjust*" OR "Diagnosis adjust*" OR "Cancer adjust*" OR "Disease adjust*" OR "Adapt* to illness" OR "Adapt* to diagnosis" OR "Adapt* to cancer" OR "Adapt* to disease" OR "Illness adapt*" OR "Diagnosis adapt*" OR "Cancer adapt*" OR "Disease adapt*" OR "Mak* peace" OR "Peace

	with illness" OR "Peace with diagnosis" OR "Peace with cancer" OR "Peace with disease" OR "Peace* accept*"))
**Cinhal**	((MH "Neoplasms+") OR (TI cancer* OR AB cancer*) OR (TI tumo#r OR AB tumo#r) OR (TI oncolog* OR AB oncolog*) OR (TI sarcoma* OR AB sarcoma*) OR (TI carcinoma* OR AB carcinoma*) OR (TI neoplas* OR AB neoplas*)) AND ((MH "Life purpose") OR (MH "Spirituality") OR (MH "Hardiness") (MH "Self Concept+") OR (TI "Purpose in life" OR AB "Purpose in life") OR (TI "Life purpose" OR AB "Life purpose") OR (TI "Sense of purpose" OR AB "Sense of purpose") OR (TI "Sense of coherence" OR AB "Sense of coherence") OR (TI "Meaning-making" OR AB "Meaning-making") OR (TI "Meaning making" OR AB "Meaning making") OR (TI "Meaning in life" OR AB "Meaning in life") OR (TI "Finding meaning" OR AB "finding meaning") OR (TI "Meaning of life" OR AB "Meaning of life") OR (TI "Sense of meaning" OR AB "Sense of meaning") OR (TI "Life meaning" OR AB "Life meaning") OR (TI "Search for meaning" OR AB "Search for meaning") OR (TI "Personal meaning" OR AB "Personal meaning") OR (TI "Global belief*" OR AB "Global belief*") OR (TI "Existential" OR AB "Existential") OR (TI "MEANINGFULNESS" OR AB "MEANINGFULNESS") OR (TI "Self-trascendence" OR AB "Self-trascendence") OR (TI "Spiritual well-being" OR AB "Spiritual well-being") OR (TI "Life engagement" OR AB "Life engagement") OR (TI "Evaluation of life" OR AB "Evaluation of life") OR (TI "Value of life" OR AB "Value of life")) AND ((MH "Adaptation, psychological+") OR ("Accept* of illness" OR "Accept* of diagnosis" OR "Accept* of cancer" OR "Accept* of disease" OR "Accept* illness" OR "Accept* diagnosis" OR "Accept* cancer" OR "Accept* disease" OR "Illness accept*" OR "Diagnosis accept*" OR "Cancer accept*" OR "Disease accept*" OR "Adjust* to illness" OR "Adjust* to diagnosis" OR "Adjust* to cancer" OR "Adjust to disease" OR "Illness adjust*" OR "Diagnosis adjust*" OR "Cancer adjust*" OR "Disease adjust*" OR "Adapt* to illness" OR "Adapt* to diagnosis" OR "Adapt* to cancer" OR "Adapt* to disease" OR "Illness adapt*" OR "Diagnosis adapt*" OR "Cancer adapt*" OR "Disease adapt*" OR "Mak* peace" OR "Peace with illness" OR "Peace with diagnosis" OR "Peace with cancer" OR "Peace with disease" OR "Peace* accept*"))
**Scopus**	INDEXTERMS (neoplasms) OR TITLE-ABS-KEY (cancer* OR tumo?r* OR oncolog* OR sarcoma* OR carcinoma*) AND INDEXTERMS (meaning) OR INDEXTERMS ("Psychological Resilience" OR spirituality OR "Self concept") OR TITLE-ABS-KEY ("Purpose in life" OR "life purpose" OR "sense of purpose" OR "sense of coherence" OR "Meaning making" OR "Meaning-making" OR "Meaning in life" OR "Finding meaning" OR "Meaning of life" OR "Personal meaning" OR "Search for meaning" OR "Life meaning" OR "Sense of meaning" OR "Spiritual well-being" OR "Self-trascendence" OR MEANINGFULNESS OR "global belief*" OR "existential" OR "life engagement" OR "evaluation of life" OR "value of life") AND INDEXTERMS (psychological AND adaptation) OR TITLE-ABS-KEY ("Accept* of illness" OR "Accept* of diagnosis" OR "Accept* of cancer" OR "Accept* of disease" OR "Accept* illness" OR "Accept* diagnosis" OR "Accept* cancer" OR "Accept* disease" OR "Illness accept*" OR "Diagnosis accept*" OR "Cancer accept*" OR "Disease accept*" OR "Adjust* to illness" OR "Adjust* to diagnosis" OR "Adjust* to cancer" OR "Adjust to disease" OR "Illness adjust*" OR "Diagnosis adjust*" OR "Cancer adjust*" OR "Disease adjust*" OR "Adapt* to illness" OR "Adapt* to diagnosis" OR "Adapt* to cancer" OR "Adapt* to disease" OR "Illness adapt*" OR "Diagnosis adapt*" OR "Cancer adapt*" OR "Disease adapt*" OR "Mak* peace" OR "Peace with illness" OR "Peace with diagnosis" OR "Peace with cancer" OR "Peace with disease" OR "Peace* accept*")

Table S2. A priori selected questionnaires to measure meaning in life.

Appraisal of Life Events (ALE) Scale [1]
Belief in an Afterlife Scale [2]
Centrality of Event Scale (CES) [3]
Constructed Meaning Scale (CMS) [4]
Core Beliefs Inventory [5]

Daily Meaning Scale (DMS) [6]
Engagement in Meaningful Activities Survey [7]
Event-Related Rumination Inventory [8] - Deliberate Processing subscale and Intrusive Ruminations subscale
Existence Scale (ES) [9]
Existential issues [10]
Existential Loneliness Questionnaire (ELQ) [11]
Existential Meaning Scale (EMS) [12]
Expressions of Life Meaning [13]
FACIT – Sp - Meaning subscale [14]
General Life Purpose Scale (GLPS) [15]
Global Goals Interview Questionnaire [16]
Global Meaning Violation [17]
Illness Perception Questionnaire-Revised (IPQ-R) [18]
Impact of Cancer (IOC) [19]
Impact of Event Scale – Intrusive thoughts subscale and Avoidance subscale (IES) [20]
Internal–External Locus of Control Scale [21]
Just World Scale-Revised [22]
Kunzendorf No Meaning and Negative Meaning Scales (NoM & NeM) [23]
Life Attitude Profile (LAP), incl. Personal Meaning Index (PMI) [24]
Life Attitude Profile- Revised – Personal Meaning Index [25]
Life Attitude Scale for Elderly with Chronic Disease (LAS) [26]
Life Engagement Test (LET) [27]
Life Esteem Survey (LES) [28]
Life Evaluation Questionnaire (LEQ) [29]
Life Goals Inventory [30]
Life Meaningfulness Scale (LMS) [31]
Life Purpose Questionnaire (LPQ) [32]
Life Regard Index and its subscales (LRI) [33]
Life Regard Index-Revised [34]
LOGO Test [35]
Major Life Goals [36]
McGill Quality of Life Scale - Existential Subscale [37]
Meaning in Life [38]
Meaning in Life (ML) [39]
Meaning in life[40]
Meaning in Life Index (MILI) [41]
Meaning in Life Questionnaire (MILQ) [42]
Meaning in Life Questionnaire and its subscales (MLQ) [43]
Meaning in Life Scale (MILS) [44]
Meaning in Life Scale[45]
Meaning in Suffering Test (MIST) [46]
Meaning-Making Scale (MMS) [47]
Meaningful Activity Participation Assessment (MAPA) [48]
Meaningful Life Measure (MLM) [49]
Measure of Mundane Meaning (MMM) [50]
Orientations to Happiness Scale – Life of meaning subscale [51]
Perceived Personal Meaning Scale (PPMS) [52]
PERMA-Profiler – Meaning subscale [53]
Personal Meaning Profile (PMP) [52]
Personal Projects Analysis [54,55]
Personal Strivings Assessment [56]
Psychological Well-Being Scales – Purpose subscale [57]
Purpose in Life Test (PIL) [58]
Quality-of-life Concerns in the End of Life – Value-of-life Subscale [59]
Questionnaire for Eudaimonic Well-Being (QEWB) [60]
Questionnaire on Meaning [61]
Revised Causal Dimension Scale (CDS-II) [62]

Ruminative Responses Inventory - Reflection subscale [63]
Search for Meaning Survey [64]
Seeking of Noetic Goals Test (SONG) [65]
Self-blame appraisal PTCI - Self-blame subscale [66]
Self-transcendence Meaning of Life Scale (STMS) [67]
Self-Transcendence Scale [68]
Sense Making Scale (SMS) [69]
Sense of Coherence Scale – Meaningfulness Subscale [70]
Situational Appraisal Measure (SAM) [71]
Sources of Life Meaning (SLM) [72]
Sources of meaning and meaningfulness questionnaire (SoMe) [73]
Sources of Meaning Profile (SOMP) [74]
Spiritual Meaning Scale (SMS) [75]
Spiritual Well-being Scale– Existential Subscale [76]
Trauma and Attachment Belief Scale [77]
Valuation of Life Scale (VOL) [78]
Views of Suffering Scale [79]
World Assumptions Scale [80]
Worldview Assessment Inventory [82]

Table S3. Acceptance of cancer measures included:

Peace, Equanimity and Acceptance in the Cancer Experience - Acceptance Subscale [82]
Peace, Equanimity and Acceptance in the Cancer Experience - Struggle Subscale [82]
Acceptance of Illness Scale [83]
Acceptance of Disability [84]
Illness Cognitions Questionnaire - Acceptance Subscale [85]
Brief COPE - Acceptance Subscale [86]
COPE - Acceptance Subscale [87]
Coping with Colorectal Cancer Scale - Acceptance Subscale [88]
Cancer Behavior Inventory - Accepting Cancer/Maintaining Positive Attitude Subscale [89]
Cancer Behavior Inventory version 2.0 - Accepting Cancer/Maintaining Positive Attitude Subscale [90]
Cancer Behavior Inventory version 3.0 - Accepting Cancer/Maintaining Positive Attitude Subscale [91]
Cognitive Emotion Regulation Questionnaire - Acceptance Subscale [92]

References

1. Ferguson, E.; Matthews, G.; Cox, T. The Appraisal of Life Events (ALE) scale: Reliability and validity. *Br. J. Health Psychol.* **1999**, *4*, 97–116.
2. Osarchuk, M.; Tatz, S.J. Effect of induced fear of death on belief in afterlife. *J. Pers. Soc. Psychol.* **1973**, *27*, 256–260.
3. Berntsen, D.; Rubin, D.C. The centrality of event scale: A measure of integrating a trauma into one’s identity and its relation to post-traumatic stress disorder symptoms. *Behav. Res.Ther.* **2006**, *44*, 219–231.
4. Fife, BL. The measurement of meaning in illness. *Soc. Sci. Med.* **1995**, *40*, 1021–1028.
5. Cann, A.; Calhoun, L.G.; Tedeschi, R.G.; Kilmer, R.P.; Gil-Rivas, V.; Vishnevsky, T.; Danhauer, S.C. (2010). The core beliefs inventory: A brief measure of disruption in the assumptive world. *Anxiety Stress Coping* **2010**, *23*, 19–34.
6. Kashdan, T.B.; Steger, M.F. Curiosity and pathways to well-being and meaning in life: traits, states, and everyday behaviors. *Motiv. Emotion.* **2007**, *31*, 159–173.
7. Goldberg, B.; Brintnell, E.S.; Goldberg, J. The relationship between engagement in meaningful activities and quality of life in persons disabled by mental illness. *Occup. Ther. Ment. Health* **2002**, *18*, 17–44.
8. Cann, A.; Calhoun, L.G.; Tedeschi, R.G.; Triplett, K.N.; Vishnevsky, T.; Lindstrom, C.M. Assessing posttraumatic cognitive processes: The event related rumination inventory. *Anxiety Stress Coping* **2011**, *24*, 137–156.
9. Längle, A.; Orgler, C.; Kundi, M. The Existence Scale: a new approach to assess the ability to find personal meaning in life and to reach existential fulfilment. *Eur. Psychother.* **2003**, *4*, 157–173.
10. la Cour, P. Existential and religious issues when admitted to hospital in a secular society: patterns of change. *Ment. Health Relig. Cult.* **2008**, *11*, 769–782.
11. Mayers, A.M.; Khoo, S-T.; Svartberg, M. The Existential Loneliness Questionnaire: background, development, and preliminary findings. *J. Clin. Psychol.* **2002**, *58*, 1183–1193.

12. Lyon, D.E.; Younger, J. Development and preliminary evaluation of the Existential Meaning Scale. *J. Holist. Nurs.* **2005**, *23*, 54–65.
13. Laverty, W.; Kelly, I.; Janzen, B.; Pringle-Nelson, C.; Miket, M. Expressions of life meaning among college students. *Psychol. Rep.* **2005**, *97*, 945–954.
14. Peterman, A.H.; Fitchett, G.; Brady, M.J.; Hernandez, L.; Cella, D. Measuring spiritual well-being in people with cancer: The functional assessment of chronic illness therapy—Spiritual Well-being Scale (FACIT-Sp). *Ann. Behav. Med.* **2002**, *24*, 49–58.
15. Byron, K.; Miller-Perrin, C. The value of life purpose: purpose as a mediator of faith and well-being. *J. Posit. Psychol.* **2009**, *4*, 64–70.
16. Klinger, E. The interview questionnaire technique: Reliability and validity of a mixed idiographic-nomothetic measure of motivation. In *Advances in personality assessment*; Butcher, J.N.; Spielberger, C.D., Eds. Lawrence Erlbaum: Hillsdale, NJ, 1987; pp. 31–48.
17. Park, C.L.; Mills, M.A.; Edmondson, D. PTSD as meaning violation: Testing a cognitive worldview perspective. *Psychol. Trauma* **2012**, *4*, 66–73.
18. Moss-Morris, R.; Weinman, J.; Petrie, K.J.; Horne, R.M Cameron, L.D.; Buick, D. The Revised Illness Perception Questionnaire (IPQ-R). *Psychol. Health* **2002**, *17*, 1–16.
19. Zebrack, B.J.; Ganz, P.A.; Bernaards, C.A.; Petersen, L.; Abraham, L. Assessing the impact of cancer: development of a new instrument for long-term survivors. *Psychooncology* **2006**, *15*, 407–421.
20. Horowitz, M.; Wilner, N.; Alvarez, W. Impact of event scale: A measure of subjective stress. *Psychosom. Med.* **1979**, *41*, 209–218.
21. Rotter, J.B. Generalized expectancies for internal versus external control of reinforcement. *Psychol. Monogr.* **1966**, *80*, 1–28.
22. Rubin, Z.; Peplau, L.A. Who believes in a just world? *J. Soc. Issues* **1975**, *31*, 65–89.
23. Kunzendorf, R.; Moran, C.; Gray, R. Personality traits and reality-testing abilities, controlling for vividness of imagery. *Imagin. Cogn. Pers.* **1995**, *15*, 113–131.
24. Reker, G.T.; Peacock, E.J. The Life Attitude Profile (LAP): a multidimensional instrument for assessing attitudes toward life. *Can. J. Behav. Sci.* **1981**, *13*, 264–273.
25. Reker, G.T. *Manual of the life attitude profile-revised*; Student Psychologists Press: Peterborough, 1992.
26. Liu, S. The construction and evaluation of the reliability and validity of a life attitude scale for elderly with chronic disease. *J. Nurs. Res.* **2001**, *9*, 33–42.
27. Scheier, M.F.; Wrosch, C.; Baum, A.; Cohen, S.; Martire, L.M.; Matthews, K.A.; Schultz, R.; Zdaniuk, B. The life engagement test: Assessing purpose in life. *J. Behav. Med.* **2006**, *29*, 291–298.
28. Wheeler, R.J.; Munz, D.C.; Jain, A. Life goals and general well-being. *Psychol. Rep.* **1990**, *66*, 307–312.
29. Salmon, P.; Manzi, F.; Valori, R.M. Measuring the meaning of life for patients with incurable cancer: the life evaluation questionnaire (LEQ). *Eur. J. Cancer* **1996**, *32*, 755–760.
30. Bower, J.E.; Kemeny, M.E.; Taylor, S.E.; Fahey, J.L. Finding positive meaning and its association with natural killer cell cytotoxicity among participants in a bereavement-related disclosure intervention. *Ann. Behav. Med.* **2003**, *25*, 146–155.
31. Halama, P. Relationship between meaning in life and the big five personality traits in young adults and the elderly. *Stud. Psychol.* **2005**, *47*, 167–178.
32. Hutzell, R.R.; Peterson, T. Use of the life purpose questionnaire with an alcoholic population. *Int. J. Addict.* **1986**, *21*, 51–57.
33. Battista, J.; Almond, R. The development of meaning in life. *Psychiatry* **1973**, *36*, 409–427.
34. Debats, D.L. Measurement of personal meaning: The psychometric properties of the life regard index. In *The human quest for meaning: A handbook of psychological research and clinical applications*; Wong, P.T.P.; Fry, P.S., Eds. Lawrence Erlbaum: Mahwah, NJ,1998; pp. 237–259.
35. Lukas, E. *LOGO-Test. Test zur Messung von "innerer Sinnerfüllung" und "existentieller Frustration"*. Deuticke: Wien, 1986.
36. Roberts, B.W.; Robins, R.W. Broad dispositions, broad aspirations: The intersection of personality traits and major life goals. *Person. Soc. Psychol. Bull.* **2000**, *26*, 1284–1296.
37. Cohen, S.R.; Mount, B.M.; Strobel, M.G.; Bui, F. The McGill Quality of Life Questionnaire: a measure of quality of life appropriate for people with advanced disease: A preliminary study of validity and acceptability. *Palliat. Med.* **1995**, *9*, 207–219.
38. Krause, N. Stressors arising in highly valued roles, meaning in life, and the physical health status of older adults. *J. Gerontol. Psychol.* **2004**, *59B*, S287–S291.
39. Warner, S.C.; Williams, J.I. The Meaning in Life Scale: determining the reliability and validity of a measure. *J. Chronic Dis.* **1987**, *40*, 503–512.
40. Tomich, P.L.; Helgeson, V.S. Five years later: a cross-sectional comparison of breast cancer survivors with healthy women. *Psychooncology* **2002**, *11*, 154–169.
41. Francis, L.J.; Hills, P.R. The development of the Meaning in Life Index (MILI) and its relationship with personality and religious behaviours and beliefs among UK undergraduate students. *Ment. Health Relig. Cult.* **2008**, *11*, 211–220.
42. Kernes, J.L.; Kinnier, R.T. Meaning in psychologists' personal and professional lives. *J. Humanist. Psychol.* **2008**, *48*, 196–220.
43. Steger, M.F.; Frazier, P.; Oishi, S.; Kaler, M. The meaning in life questionnaire: Assessing the presence of and search for meaning in life. *J. Couns. Psychol*. **2006**, *53*, 80–93.
44. Jim, H.S.; Purnell, J.Q.; Richardson, S.A.; Golden-Kreutz, D.; Andersen, B.L. Measuring meaning in life following cancer. *Qual. Life Res.* **2006**, *15*, 1355–1371.

45. Thompson, S.C.; Sobolew-Shubin, A.; Graham, M.; Janigian, A. Psychosocial adjustment following a stroke. Soc. Sci. Med. **1989**, *28*, 239–247.
46. Starck, P.L. Patients' perceptions of the meaning of suffering. *Int. Forum Logotherapy* **1983**, *6*, 110–116.
47. van den Heuvel, M.; Demerouti, E.; Schreurs, B.H.J.; Bakker, A.B.; Schaufeli, W.B. Does meaning-making help during organizational change?: development and validation of a new scale. *Career Develop. Inter.* **2009**, *14*, 508–533.
48. Eakman, A.M.; Carlson, M.E.; Clark, F.A. The Meaningful Activity Participation Assessment: a measure of engagement in personally valued activities. *Int. J. Aging Hum. Dev.* **2010**, *70*, 299–317.
49. Morgan, J.; Farsides, T. Measuring meaning in life. *J. Happiness Stud.* **2009**, *10*, 197–214.
50. Brown, G.P.; Roach, A.; Irving, L.; Joseph, K. Personal meaning: a neglected transdiagnostic construct. *Int. J. Cogn. Ther.* **2008**, *1*, 223–236.
51. Peterson, C.; Park, N.; Seligman, M.E.P. Orientations to happiness and life satisfaction: the full versus the empty life. *J. Happiness Stud.* **2005**, *6*, 25–41.
52. Wong, P.T.P. Implicit theories of meaningful life and the development of the Personal Meaning Profile. In *The Human Quest for Meaning. A handbook of Psychological Research and Clinical Applications*, Wong, P.T.P.; Fry, P.S., Eds. Lawrence Erlbaum: Mahwah, NJ,1998; pp. 111–140.
53. Butler, J.; Kern, M.L. The PERMA-profiler: A brief multidimensional measure of flourishing. *Int. J. Wellbeing* **2016**, *6*, 1–48.
54. Little, B.R. Personal projects: A rationale and method for investigation. *Environ. Behav.* **1983**, *15*, 273–309.
55. Little, B.R.; Gee, T.L. (2007). The methodology of personal projects analysis: Four modules and a funnel. In *Personal project pursuit: Goals, action, and human flourishing*, Little, B.R.; Salmela-Aro, K.; Phillips, S.D., Eds. Lawrence Erlbaum: Mahwah, NJ, 2007; pp. 51-94.
56. Emmons, R.A. *The psychology of ultimate concerns: Motivation and spirituality in personality*. New York, NY: Guilford Press; 1999.
57. Ryff, C.D. Happiness is everything, or is it? Explorations on the meaning of psychological well-being. *J. Pers. Soc. Psychol.* **1989**, *57*, 1069–1081.
58. Crumbaugh, J.C.; Maholick, L.T. An experimental study in existentialism: The psychometric approach to Frankl's concept of noogenic neurosis. *J. Clin. Psychol.* **1964**, *20*, 200–207.
59. Pang, S.M.; Chan, K.S.; Chung, B.P.; Lau, K.S.; Leung, E.M.; Leung, A.W.; Chan, H.; & Chan, T.M. Assessing quality of life of patients with advanced chronic obstructive pulmonary disease in the end of life. *J. Palliat. Care* **2005**, *21*, 180–187.
60. Waterman, A.S.; Schwartz, S.J.; Zamboanga, B.L.; Ravert, R.D.; Williams, M.K.; Agocha, V.B.; Yeong Kim, S.; Donnellan, B. The Questionnaire for Eudaimonic Well-Being: psychometric properties, demographic comparisons, and evidence of validity. *J. Posit. Psychol.* **2010**, *5*, 41–61.
61. Wheeler, I. The role of meaning and purpose in life in bereaved parents associated with a self-help group: compassionate friends. *J. Death Dying* **1993**, *28*, 261–271.
62. McAuley, E.; Duncan, T.E.; Russell, D.W. Measuring causal attributions: The revised Causal Dimension Scale (CDSII). *Pers. Soc. Psychol. Bull.* **1992**, *18*, 566–573.
63. Treynor, W.; Gonzalez, R.; Nolen-Hoeksema, S. (2003). Rumination reconsidered: A psychometric analysis. *Cogn. Ther. Res.* **2003**, *27*, 247–259.
64. Taylor, E.J. Factors associated with meaning in life among people with recurrent cancer. *Oncol. Nurs. Forum* **1993**, *20*, 1399–1407.
65. Crumbaugh, J.C. The Seeking of Noetic Goals Test (SONG): a complementary scale to the Purpose in Life Test (PIL). *J. Clin. Psychol.* **1977**, *33*, 900–907.
66. Foa, E.B.; Ehlers, A.; Clark, D.M.; Tolin, D.F.; Orsillo, S.M. The Posttraumatic Cognitions Inventory (PTCI): Development and validation. *Psychol. Assess.* **1999**, *11*, 303–314.
67. Li, H. College stress and psychological well-being: vision in life as a coping resource. Doctoral dissertation, University of Hong Kong, Hong Kong, 2002.
68. Haugan, G.; Rannestad, T.; Garåsen, H.; Hammervold, R.; Espnes, G.A.The self-transcendence scale: an investigation of the factor structure among nursing home patients. *Journal of Holist. Nurs.* **2012**, *30*, 147–159.
69. Pakenham, K.I. Making sense of multiple sclerosis. *Rehabil. Psychol.* **2007**, *52*, 380–389.
70. Antonovsky, A. The structure and properties of the sense of coherence scale. *Soc. Sci. Med.* **1993**, *36*, 725–733.
71. Peacock, E.; Wong, P.T.P. The Stress Appraisal Measure (SAM): A multidimensional approach to cognitive appraisal. *Stress Med.* **1990**, *6*, 227–236.
72. Prager, E. Exploring personal meaning in an age-differentiated Australian sample: another look at the Sources of Meaning Profile (SOMP). *J. Aging Stud.* **1996**, *10*, 117–136.
73. Schnell, T. The Sources of Meaning and Meaning in Life Questionnaire (SoMe): Relations to demographics and well-being. *J. Posit. Psychol.* **2009**, *4*, 483– 499.
74. Reker, G.T.; Wong, P.T.P. (1988). Aging as an individual process: Toward a theory of personal meaning. In Emergent theories of aging, Bengtson, I.J.E.B., Ed. Springer: New York, NY, 1988; pp. 214–246.
75. Mascaro, N.; Rosen, D.H.; Morey, L.C. The development, construct validity, and clinical utility of the spiritual meaning scale. *Pers. Individ. Diff.* **2004**, *37*, 845–860.
76. Ellison, C.W. Spiritual well-being: Conceptualization and measurement. *J. Psychol. Theol.* **1983**, *11*, 330–338.
77. Pearlman, L.A. *Trauma and Attachment Belief Scale (TABS).* Los Angeles, CA: Western Psychological Services; 2003.

78. Lawton, M.P.; Moss, M.; Hoffman, C.; Kleban, M.H.; Ruckdeschel, K.; Winter, L. Valuation of life: a concept and a scale. *J. Aging Health* **2001**, *13*, 3–31.
79. Hale-Smith, A.; Park, C.L.; Edmondson, D. Measuring beliefs about suffering: Development of the views of suffering scale. *Psychol. Assess.* **2012**, *24*, 855–866.
80. Janoff-Bulman, R. Assumptive worlds and the stress of traumatic events: Applications of the schema construct. *Soc. Cogn.* **1989**, *7*, 113–136.
81. Koltko-Rivera, M. (2000). The Worldview Assessment Instrument (WAI): The development and preliminary validation of an instrument to assess world view components relevant to counseling and psychotherapy. Doctoral dissertation, School of Education -New York University, New York, NY, 2020.
82. Mack, J.W.; Nilsson, M.; Balboni, T.; Friedlander, R.J.; Block, S.D.; Trice, E.; Prigerson, H.G. Peace, Equanimity, and Acceptance in the Cancer Experience (PEACE): Validation of a scale to assess acceptance and struggle with terminal illness. *Cancer* **2008**, *112*, 2509–2517
83. Felton, B.J.; Revenson, T.A.; Hinrichsen, G.A. Stress and coping in the explanation of psychological adjustment among chronically ill adults. *Soc. Sci. Med.* **1984**, *18*, 889–898.
84. Linkowski, D.C. A scale to measure acceptance of disability. *Rehabil. Couns. Bull.* **1971**, *14*, 236–244.
85. Evers, A.W.; Kraaimaat, F.W.; van Lankveld, W.; Jongen, P.J.; Jacobs, J.W.; Bijlsma, J.W. Beyond unfavorable thinking: the illness cognition questionnaire for chronic diseases. *J. Consult. Clin. Psychol.* **2001**, *69*, 1026–1036.
86. Carver, C.S. You want to measure coping but your protocol's too long: Consider the Brief COPE. *Int. J. Behav. Med*. **1997**, *4*, 92–100.
87. Carver, C.S.; Scheier, M.F.; Weintraub, J.K. Assessing coping strategies: A theoretically based approach. *J. Pers. Soc. Psychol.* **1989**, *56*, 267–283.
88. Rinaldis, M.; Pakenham, K.I.; Lynch, B.M.; Aitken, J.F. Development, confirmation, and validation of a measure of coping with colorectal cancer: a longitudinal investigation. *Psychooncology* **2009**, *18*, 624–633.
89. Merluzzi, T.V.; Martinez Sanchez, M.A. Assessment of self-efficacy and coping with cancer: development and validation of the cancer behavior inventory. *Health Psychol.* **1997**, *16*, 163–170.
90. Merluzzi, T.V.; Nairn, R.C.; Hegde, K.; Martinez Sanchez, M.A.; Dunn, L. Self-efficacy for coping with cancer: revision of the Cancer Behavior Inventory (version 2.0). *Psychooncology* **2001**,*10*, 206-217.
91. Merluzzi, T.V.; Philip, E.J.; Heitzmann Ruhf, C.A.; Liu, H.; Yang, M.; Conley, C.C. Self-efficacy for coping with cancer: Revision of the Cancer Behavior Inventory (Version 3.0). *Psychol. Assess.* **2018**, *30*, 486–499.
92. Garnefski, N.; Kraaij, V. The cognitive emotion regulation questionnaire. *Eur. J. Psychol. Assess.* **2007**, *23*, 141–149.
